# Solitary rectal ulcer syndrome: addition of rectal therapies to biofeedback is more effective than biofeedback alone 

**Published:** 2019

**Authors:** Saeed Abdi, Narjes Tavakolikia, Mehdi Yamini, Mohammad Bagheri, Amir Sadeghi, Mohamad Amin Pourhoseingholi, Shabnam Shahrokh, Morteza Aghajanpoor Pasha

**Affiliations:** 1 *Gastroenterology and Liver Diseases Research Center, Research Institute for Gastroenterology and Liver Diseases, Shahid Beheshti University of Medical Sciences, Tehran, Iran*; 2 *Departments of Sociocultural, Tehran University of Medical Sciences, Tehran, Iran*

**Keywords:** Biofeedback, SRUS, Endoscopic responses

## Abstract

**Aim:**

We designed this study to evaluate the effectiveness of the combination of topical rectal therapy with biofeedback in treatment of solitary rectal ulcer compared to single biofeedback therapy.

**Background:**

Biofeedback therapy is an appropriate treatment for patients with solitary rectal ulcer syndrome (SRUS) but it seems that it is not effective alone. Topical medical therapies are supposed to have an additive role to biofeedback.

**Methods:**

This randomized, controlled trial was conducted on 63 patients with SRUS. Patients were randomly enrolled into two groups of combination and single therapy. The patients in combination group (n=31) received biofeedback plus a topical therapy (an enema contained dexamethasone, sulfasalazine and bismuth) and the patients in single therapy group (n=32) were treated with biofeedback alone.

**Results:**

Endoscopic responses to treatment in the combination and single groups were 80% and 50%, respectively (P<0.05). Clinical improvement in symptoms such as difficulty to evacuate, digitation to evacuate, feeling of incomplete evacuation, time to need to evacuation and life style alternation were significantly better in treated group by combination therapy than single therapy. Regarding to the mean total score based on all subjective parameters, the results were also significantly better in the treated group by combination therapy.

**Conclusion:**

Topical anti-inflammatory therapies in combination with biofeedback is an efficient treatment for patients with SRUS

## Introduction

 Solitary rectal ulcer syndrome (SRUS) is an uncommon benign disorder characterized by single or multiple ulcerations of the rectal mucosa, with the passage of blood and mucus, associated with straining or abnormal defecation (1, 2). Clinical features include rectal bleeding, copious mucus discharge of rectum associated with abdominal pain, prolonged excessive straining, feeling of incomplete defecation, constipation, and rarely rectal prolapse (3). The pathogenesis of SRUS is not well understood, but may be multifactorial (4). The most accepted theory of the mechanism of the SRUS is related to direct trauma or ischemic injury (5). The etiology is associated with paradoxical contraction of the pelvic floor leading to mucosal prolapse and pressure necrosis of rectal mucosa (6). Another hypothesis suggests that external anal sphincter produces abnormal pressure gradients in the opposite direction which results in abnormal defecation leading to SRUS (7). Due to wide range of clinical symptomatology and endoscopic findings, SRUS may often simulate other disorders such as inflammatory bowel disease (IBD) and neoplasm (8). 

Several treatment options have been used for SRUS, ranging from conservative treatment (i.e., diet and bulking agents), medical therapy, biofeedback and surgery.(9) The SRUS may relate to chronic straining in some patients. Therefore, an organized form of behavior therapy such as biofeedback appears to be promising (10). Behavioral modification or biofeedback therapy improves both rectal blood flow and symptom, this treatment also includes bowel habit training, avoiding excessive straining, and normalization of pelvic floor coordination (11, 12). In a previous study, standard biofeedback therapy improved both anorectal function and bowel symptoms in most patients who exhibited dissynergic defecation (13, 14). In addition, medical treatments such as sucralfate, salicylate, corticosteroids, sulfasalazine, mesalazine and topical fibrin sealant, have been reported to be effective with various responses and improvement of symptoms (15). Corticosteroids and sulfasalazine enemas may also help ulcer healing by reducing the inflammatory responses (16, 17). Also, bismuth enema can be an effective option in rectal diseases (18).

Because of the very favorable risk/benefit ratio and the low rate of adverse effects of corticosteroids and sulfasalazine enemas on SRUS, we made a decision to use these as an anti-inflammatory supplement with biofeedback therapy to improve or modify SRUS symptoms. The purpose of this study was to evaluate the effectiveness of combination of topical rectal therapy with biofeedback in treatment of solitary rectal ulcer compared with single biofeedback therapy. 

## Methods

**Patients**

This study is a randomized controlled trial. we enrolled patients that were diagnosed as SRUS who referred to Taleghani hospital in 2017-2018. SRUS of all subjects was diagnosed by anorectal manometry and colonoscopy. Demographic data such as age, gender, clinical symptoms and colonoscopy, histology and manometry findings were evaluated in all patients. Patients who were taking drugs which make constipation were excluded. Written informed consent was obtained from all patients and the study was approved by the Ethics Committee of the Research Institute for Gastroenterology and Liver Diseases, Shahid Beheshti University of Medical Sciences, Tehran, Iran.

**Study design**

In this randomized controlled trial study, 63 subjects with diagnosed SRUS were satisfied to participate in this study. Patients with SRUS were randomly assigned into two groups: combination therapy of biofeedback plus topical therapy (Group A) and single biofeedback therapy (Group B). In group A (n=31), patients received an enema contained dexamethasone (1 mg), bismuth (2 gr) and sulfasalazine (2 gr) daily for 4 months. Group B (n=32), only used a single biofeedback treatment; Biofeedback therapy involves more than just retraining pelvic floor co-ordination (11). It also teaches patients the necessary posture and use of abdominal muscles during defecation, and imposes a discipline about number of visits to the toilet, time spent in the toilet, digitation, and laxative use (12). The phase of biofeedback therapy consisted of four months with eight sessions of biofeedback, which all patients were provided with education and medical management. The duration of training visits was the same for patients in all two groups (approximately 60 minutes). Education included information on the physiology of defecation and the normal range of bowel movements, including a review of their manometry test results and use of drawings of pelvic floor anatomy to illustrate the concept of pelvic floor dyssynergia. Patients were instructed to keep diaries, recording unassisted bowel movements, assisted bowel movements, straining, and the feeling of incomplete evacuation. In addition, patients should receive instructions regarding timed toilet training and laxatives. Timed toilet training consists of educating the patient to attempt a bowel movement at least twice a day, usually 30 minutes after meals and to strain for no more than 5 minutes (10, 19). Patients also received recommendations regarding changes in diet (increased fiber), water/diuretic intake, and general physical exercise to improve stool consistency (lifestyle alternation). All patients were visited every two weeks in duration of treatment.

**Measurement**

All patients were visited every two weeks in duration of treatment and follow-up. Evaluation was performed after execution of the procedures. Outcomes were assessed at baseline and after treatment. Before and after intervention, the colonoscopy findings were evaluated by the expert gastroenterologists and the percent of treatment was determined in colonoscopy. In all endoscopy sessions, the characteristics of ulcer, ulcer size, number of ulcers, and stigmata of bleeding were recorded during the session, and the course of treatment was evaluated accordingly. All symptoms such as medication to evacuation (enema or suppositories), time needed to evacuation, difficulties to evacuate, digitation to evacuate, life style alteration, return to toilet to evacuate, straining to evacuation, feeling of incomplete evacuation and rectal bleeding were assessed before and after treatment in two groups with self-report in scaling (zero was lowest and three was highest score).

**Statistical analysis**

Statistical Package for the Social Sciences (SPSS) 21.0 (Chicago, IL, USA) was used for analysis. Comparison of variables was performed using Pearson’s chi-square test, Fisher’s exact test, or t-test, depending on the nature of the data. Comparing the changes in symptoms (score) before and after treatment was assessed by Wilcoxon Signed Ranks Test. difference between Total mean score of two groups which used to compare the efficacy of treatment, was analyzed by independent sample t-test and Mann-Whitney U. P-value <0.05 considered to be statistically significant. 

## Results

**Patient’s demographic characteristics**

The present study recruited 63 patients with SRUS; 33 (52.4%) female and 30 (47.6%) male. All subjects were randomly divided into two groups; group A (n=31) who received combination therapy and group B (n=32) received a single therapy of biofeedback. The age (Mean ± SD) of all participants was 38.5±11.6 years, with no significant difference between two groups (40.2±10.1 vs. 36.3±12.6, P=0.11). 

Demographic characteristics of patients are summarized in Table 1. To insure that the groups were not different prior to treatment, they were compared by t-test analysis with respect to demographic variables and subjective parameters such as medication consumption score, difficulties of evacuation, needing digital assistance for stooling, straining effort, time of evacuation, feeling of incomplete evacuation and frequency of bowel movements. Two groups were consistent with each other and there was no significant difference between them before treatment (P>0.05) (data not shown). 

**Outcome Measures**

The mean frequency of all criteria such as medication consumption score, difficulties of evacuation, needing digital assistance for stooling, straining effort, time of evacuation and feeling of incomplete evacuation was significantly different in pre-and post-treatment evaluation in each group (P<0.001). Mean (SD) scores for symptoms before and after treatment in both groups are presented in table 2. Histological examination was considered at baseline and after treatment in both groups and regarding to the average percent of improvement in colonoscopy evaluation on all subjective parameters, the results were significantly better in group A who treated by combination therapy (80% vs. 50%), (Mean±SD: 72.17±19.1 vs. 56.09±23.4, P=0.005) than the group B who treated by a single biofeedback therapy. 

The mean frequency of all criteria such as medication consumption score, difficulties to evacuate, needing digital assistance for stooling, straining effort, time of evacuation, feeling of incomplete evacuation, lifestyle alteration and rectal bleeding was significantly different in pre-and post-treatment evaluation in each group (P<0.001). After treatment, the mean frequency of difficulty to evacuate (P=0.03), digitation to evacuate (P=0.04), feeling of incomplete evacuation (P=0.01), time to need to evacuation (P=0.009) and lifestyle alternation (P=0.01) decreased significantly in the group A, who received the combination therapy. There was no significant difference between two groups in order to reduce degree use of medication (P=0.24), return to toilet (P=0.26), straining to evacuation (P=0.12), and rectal bleeding (P=0.21). Regarding to the mean total score based on all subjective parameters, the results were significantly better in the treated group by combination therapy (7.28±2.6 vs. 10.4±3.8, P=0.001).

**Table 1:** Demographic characteristics

		Age		Gender		Total
		Mean±SD	Range	Male (%)	Female (%)	
Group A	Combination therapy	40.2±10.1 a	20-57	15 (48.4)^ a^	16 (51.6)	31 (100)
Group B	Single biofeedback	36.3±12.6	16-58	15 (49.6)	17 (53.1)	32 (100)
All participants		38.05±11.6	16-58	30 (47.6)	33 (52.4)	63 (100)

a Age and gender were not significantly different between two groups, P >0.005

**Table 2:** Effects of treatments on outcome parameters (Mean ± SD) before and after of intervention in both groups

Outcome parameters	Combination therapy			Single therapy			P-value b
	Baseline	After	P-value a	Baseline	After	P-value a	
Meditation to evacuation	2.52±0.6	1.23±0.99	<0.001	2.47±0.8	1.47±0.6	<0.001	0.246
Difficulties to evacuate	2.48±0.5	1.03±0.5	<0.001	2.34±0.6	1.31±0.5	<0.001	0.033*
Digitation to evacuate	2.39±0.6	0.68±0.6	<0.001	2.16±0.6	1.03±0.7	<0.001	0.049*
Return to toilet to evacuate	2.32±0.7	0.81±0.6	<0.001	1.63±1.15	1.03±0.93	<0.001	0.262
Feeling of incomplete	2.48±0.5	0.87±0.5	<0.001	2.13±0.6	1.29±0.8	<0.001	0.015*
Straining to evacuation	2.55±0.5	1±0.7	<0.001	2.23±0.7	1.27±0.6	<0.001	0.128
Time needed to evacuate	2.39±0.5	1.03±0.5	<0.001	2.41±0.6	1.41±0.6	<0.001	0.009*
Lifestyle alteration	2.65±0.5	0.55±0.6	<0.001	1.94±0.8	0.94±0.6	<0.001	0.012*
Rectal bleeding	1.97±0.6	0.68±0.5	<0.001	2.09±0.6	0.88±0.7	<0.001	0.219
Mean total Score	21.84±2.01	7.58±2.6	<0.001	19.28±3.5	10.4±3.8	<0.001	0.001*

a P-value between each group at baseline and after treatment,

b P-value between two groups after treatment,

* P <0.05 (statistically significant)

## Discussion

The results of this randomized trial study showed that the evaluation of the treatment via colonoscopy in the combination therapy group was significantly better than single therapy with biofeedback. The efficacy of the combination therapy was highly superior to single therapy of biofeedback with respect to the symptomatic changes such as difficulty to evacuate, digitation to evacuate, feeling of incomplete evacuation, time to need to evacuation, lifestyle alternation and also average percent of improvement in colonoscopy. The proportion of patients reporting adequate relief of constipation was significantly greater for the group receiving combination therapy compared with patients treated with only single biofeedback therapy. However, there was no statistically significant difference between two groups in terms of rectal bleeding using medication to evacuate, return to toilet to evacuate and straining to evacuate. 

 Previous studies have suggested that biofeedback is an appropriate and useful treatment for most patients with SRUS and an appropriate result has been achieved. Also, this kind of therapy increased rectal mucosal blood flow (1, 13, 14). A recent study declared that at least 50% improvement in manometry parameters obtained after biofeedback and notable improvement in symptoms after biofeedback occurred in abdominal pain and incomplete evacuation (20). Similar results acquired in our study. Another study on the role of biofeedback on SRUS concluded that biofeedback improved bowel symptoms, bowel satisfaction score and anorectal function which was similar to our results (13). However, problems have also been addressed for this treatment. Therefore, the lower number of patients can be completely treated with this type of treatment, which leads to failure of treatment (21). In addition, over time, the effects of this type of treatment may be reduced in some patients (22, 23). It has no long-term efficacy, and therefore may be needed to repeat treatment. The results of these studies suggest that these treatments do not result in complete healing (12). Therefore, beside of biofeedback therapy, medical treatment includes sucralfate, salicylate, corticosteroid, botulinum toxin, sulfasalazine and bismuth enema was used as the alternative treatment (3). Rectal corticosteroids have become popular in recent years as a treatment in ulcerative colitis and also are used to help relieve swelling, itching, and discomfort of some other rectal problems, including hemorrhoids and inflammation of the rectum caused by radiation (24, 25). Former studies suggest that intra-rectal steroids are an efficient treatment for rectal problems such as ulcerative colitis (UC) and inflammatory bowel disease (IBD) (26, 27). On the other hand, sulfasalazine consists of 5-aminosalicylic acid (5-ASA) and sulfapyridine, which are bound together by an azo bond. It has both anti-inflammatory (5-ASA) and antibacterial (sulfapyridine) properties (28). 

Corticosteroid and sulfasalazine enemas have been reported to be effective on SRUS with various responses and improvement of symptoms. To our knowledge, limited studied have been done in this field. So, long-term benefits and colonoscopic changes in rectal treatments deserves further investigation (1, 4, 29). In addition, bismuth enema is known to have a toxic effect on some microorganisms and its therapeutic benefit in rectal problems and UC may be related to this (18, 30). In a prospective study by Ryder *et al*., fifteen patients with UC which were unresponsive to conventional therapy were treated with enemas containing bismuth subsalicylate (700 or 800 mg) and showed that rectal bismuth subsalicylate appears likely to be an effective therapy in UC (30). A recent study on different treatment options of SRUS declared that multimodal study comprised of conservative, medical and surgical treatments is the best option of managing this syndrome. Better therapeutic effects in combination of medical treatment and biofeedback are in concordance with this study suggestion (31).

A limitation of this study is that we could not follow-up our patients and cannot reliably comment on the stability of our interventions over time. Despite, to our knowledge, this paper is the first research that used the corticosteroid enema plus bismuth and sulfasalazine enema with biofeedback as an anti-inflammatory supplement to achieve better efficacy in patients with SRUS. Therefore, further studies are needed to prove these findings and their long-term benefits deserve further investigation.

In conclusion; the superiority of combination therapy of biofeedback with corticosteroid (dexamethasone) plus sulfasalazine and bismuth enema were also shown by multiple outcome variables that were derived from symptomatic changes of constipation. Patients in the combination group to compare with patients, who received a single therapy of biofeedback, reported more unassisted bowel movements, simple stool without any difficulty and fewer laxative assisted bowel movements at the end of treatment. Undoubtedly, further relief of these symptoms is due to the anti-inflammatory responses of corticosteroids and sulfasalazine enemas. Corticosteroids are, together with sulfasalazine and bismuth three of the most important drugs for treatment of active SRUS as UC. The present results confirmed previous findings that corticosteroids and sulfasalazine with bismuth enema in combination with biofeedback is an efficient treatment for rectal problems and have benefits upon biofeedback therapy alone.
